# Operationalizing Good Intentions: How Organizations Are Translating Evidence Into Health Equity Action

**DOI:** 10.1016/j.jacadv.2024.100955

**Published:** 2024-05-01

**Authors:** Delaine Teabout Thomas, Antoine Keller, Joneigh S. Khaldun, Mosi Bennett, Courtney Jordan Baechler

**Affiliations:** a Minneapolis Heart Institute Foundation, Minneapolis, Minnesota, USA; b Heart and Vascular Center at Ochsner Lafayette General Hospital, Lafayette, Louisiana, USA; c CVS Health Corporation, Woonsocket, Rhode Island, USA; d Minneapolis Heart Institute, Minneapolis, Minnesota, USA

**Keywords:** health equity, prevention, translational research

As researchers, we are privileged to stand in front of the window of society observing the state of health, particularly related to cardiovascular outcomes. As we do so, we witness the unequal distribution of health, a society that exposes some to health-impacting discrimination and exclusion, and a health care workforce that does not reflect the diversity within our society. We see the impact of these social conditions including disparate access to life-saving therapies,^1^ higher prevalence of risk factors along racial and socioeconomic lines,^2^ and lack of representation in clinical trials among ethnic and gender populations most affected by cardiovascular disease (CVD).^3^^,^^4^ Three years have passed since *JACC* released its watershed Race, Ethnicity, and Cardiovascular Disease: JACC Focus Seminar Series. The anthology of 9 narrative assessments detailed cardiovascular conditions, how they are influenced by socioeconomic factors, and provided suggestions on how to work to achieve health equity. Although there has been an increase in calls-for-papers and literature describing cardiovascular disparities and evidence of their relationship to social drivers of health, little is known about how health systems, research institutions, and other entities address social drivers to achieve improved cardiovascular outcomes. The following paper aims to highlight opportunities to move beyond rhetoric and toward action by presenting examples of health equity operationalization and providing a clear call-to-action.

## Heart-sense structural heart disparities program

CVD continues to be the leading cause of death for people who identify as Black or African Americans in the United States.^5^ With the advent of transcatheter treatment for structural heart disease (SHD), significant progress has been made reducing CVD rates in patients with aortic stenosis. Recent data would suggest that Black/African Americans enjoy equivalent expectations of favorable outcomes from transcatheter aortic valve replacement,^6^ however, closer analysis reveals that Black/African Americans only comprise 4% of the treatment population while they make up 14% of the national population.^7^ These data intimate that Black/African Americans do not receive transcatheter aortic valve replacement procedures at rates commensurate with their proportion in the population, and hesitancy to participate in clinical trials has been a common explanation for this disparity, but cultural and social determinants have also been found to play a part.^8^ Current literature that informs the estimation of the prevalence of aortic stenosis in this country has determined that Black/African Americans are a significantly lower risk of developing severe aortic stenosis than White people. Further analysis would reveal that these estimations have been based upon incidence data, and retrospective extrapolation using Census data, which is inherently biased.

To determine the “true prevalence” of SHD in underserved communities, the Heart-Sense Structural Heart Disparities Program has deployed and modeled leveraging inexpensive, portable, artificial intelligence technology into at-risk communities where there is limited medical expertise. Reaching individuals that, by virtue of cultural, social, geographic, systemic, or institutional impediments, do not receive regular medical care for chronic cardiovascular conditions, our understanding of the actual prevalence of SHD has been found to be much higher than previous estimates have suggested.

With the realization that SHD prevalence may be higher in underserved communities, a focused effort for early diagnosis and treatment of individuals who may have health care access-related challenges will be essential to impacting the death rate from undiagnosed CVD in large segments of our population. Recent screening efforts for SHD in these communities have focused on echocardiographic screening which is expensive, requires technical expertise, and is time-consuming, thus limiting the number of individuals that can be evaluated in a certain time frame. The Heart-Sense program has provided a model for screening that leverages artificial intelligence technology to obtain actionable information that can give nonphysician community health advocates the confidence to refer individuals with SHD to a medical system. Using inexpensive, portable diagnostic technology and developing relationships with existing community health networks, a critical pathway to health system introduction has enhanced the likelihood of health equity related to SHD in underserved and rural communities in Louisiana. The capacity to employ nontraditional methods to discover large populations of individuals who have chronic cardiovascular problems is necessary and will save many lives and reduce health care costs by decreasing the incidence of irreversible congestive heart failure due to undiagnosed chronic disease.

## CVS health and the community equity alliance

CVS Health is dedicated to the advancement of health equity for its colleagues, consumers, clients, and communities by prioritizing trust building, increasing access to care, and by improving quality of health services and delivery in partnership with historically marginalized communities. This is accomplished through a robust strategy focused on empowering its colleagues, leveraging powerful population-based data insights, and taking bold strategic action in collaboration with community stakeholders.

One significant aspect of CVS Health's strategy for health equity involves community partnerships, particularly anchor institutions within cities such as Detroit, Nashville, and Chicago. CVS Health collaborates on addressing social determinants of health in multifaceted approaches which are meaningful for community beyond traditional research endeavors. In January 2023, CVS Health launched the Community Equity Alliance to enhance connections between health care institutions and communities, expand the community health worker (CHW) workforce, and address disparities in heart and mental health. Meharry Medical College, Sinai Chicago, and Wayne State University are the first partner institutions. The initiative utilizes the totality of CVS Health’s reach to build relationships with community partners who are important providers of services in historically marginalized communities. Investments in the CHW pipeline—a key lever to connect communities to care and build trust—underscore a corporate commitment to take bold actions that help eliminate disparities in historically marginalized communities and expand access to care. In 2023, Meharry Medical College used its Community Equity Alliance funding to successfully collaborate with health systems and academic institutions across Tennessee to recruit, train, and certify 41 new CHWs. Training consisted of an evidenced-based curriculum that included core competency training and a 40-hour field experience at a rural or urban hospital setting. More than half of these individuals also completed a 20-hour comprehensive heart health module to enhance their patient navigation skills, connect patients to care (telehealth, patient follow-up, completion of medical insurance applications, food insecurity, transportation, housing), and provide informal counseling and emotional support.

## Minneapolis heart institute foundation

The Minneapolis Heart Institute Foundation (MHIF) is committed to creating a world without heart and vascular disease for all individuals by identifying disparities, fostering trust, and building partnerships with diverse communities. The murder of George Floyd, occurring near MHIF, prompted a profound reflection on how the organization might best collaborate with communities to achieve health equity. In 2020 to 2021, MHIF embarked on deep community listening, revealing the imperative to develop culturally specific outreach and education initiatives, address mental health and its cardiovascular impact, and create opportunities for both health *and* social equity.

Internally, MHIF implemented several measures to enhance health equity awareness and action. A health equity journal club was initiated to facilitate a nuanced understanding of disparities among staff. The organization decentralized health equity work, making it a strategic priority across the entire organization, and resourced the work by introducing a groundbreaking Women’s Health Equity Fellowship focused on community-based participatory research, population health, and addressing health disparities affecting women and other marginalized populations. Externally, MHIF engaged in pipeline development by collaborating with the local community pillar Power of People Leadership Institute to develop a micro-credential for Black/African Americans high school girls interested in health careers. Currently being expanded to reach more students across the State of Minnesota, this initiative showed improvements in cardiovascular health literacy, provided exposure to cardiology professionals, and saw an increased interest in pursuing a professional path in cardiology.

MHIF also prioritized outreach and education within the Black/African-American community, forming a deep and long-term partnership with InsightNews, 1 of the first Black/African-American news publications in Minnesota. This collaboration produced the Heart-To-Heart podcast, centering Black/African-American patient voices and serving as a tool to demystify cardiac events, providing insights into how individuals navigate diagnoses, insurance, and lifestyle modifications. Again, mental health emerged as a key focus area. MHIF partnered with community leader Chance York, CVS Health, and local community members to research how mindfulness and meditation may positively impact blood pressure, quality of life, and a sense of belonging. MHIF's multifaceted approach, encompassing internal initiatives, external partnerships, pipeline development, outreach and education, mental health focus, and ongoing research studies, exemplifies its dedication to advancing health equity and eliminating cardiovascular disparities in diverse communities.

## A call to take 6 actions

What can researchers, public health, and health care professionals do today to operationalize the good intentions of working toward health equity in their practices and communities? The following lessons have been gleaned from MHIF, CVS, and Heart-Sense:

1. **Acknowledge research biases**: To effectively design interventions for health equity, it is crucial to acknowledge and address biases in current literature that contribute to prevalence estimations for disparate clinical issues among underserved communities. Specifically, recognizing the potential underestimation of risks will allow stakeholders to design more accurate and more equitable initiatives, which will likely highlight the unique needs of populations historically marginalized in health care and the greater society.
2. **Establish a firm and clear baseline**: To develop interventions that accurately and sustainably address long-standing inequities, we must develop accurate clinical and social baselines. In doing so, we may develop *realistic* impact goals, maintain a systems view of the socioeconomic factors that reify inequities and use best practices and standards to carefully address barriers to equity.
3. **Develop dynamic data collection and analysis**: The axiom “you cannot impact what you do not measure” underscores the importance of data collection and analysis which is both dynamic and responsive. Achieving consistency in data collection, assessing data quality, establishing consistency in data collection, and assessing the completeness and quality of data (eg, bias, patterning, etc) are all critical steps in ensuring our goal of equity is comprehensively achieved.
4. **Prioritize community engagement**: Data can often become most actionable when examined through the lens of local drivers of health disparities. Prioritizing community engagement and considering the lived experiences of those impacted serves to provide a critical lens on how to leverage the data and tailor the action steps needed to improve outcomes in ways that are culturally specific and aligned with the interests of said community.
5. **Invest in partnerships and collaboration**: Collaboration is key to strengthening the effectiveness and sustainability of data-driven equity solutions. Establishing multisector partnerships with local organizations, health care providers, and community leaders creates a collaborative ecosystem that pools expertise, optimizes resources, and builds trust.
6. **Bridge opportunity gaps**: A commitment to a future where cardiology reflects the diversity of the patient population necessitates the development of a pipeline to cardiology today. Investment in training and outreach programs focused on racial and gender communities historically marginalized is essential to ensuring more inclusive representation in the cardiology field, promoting trust and safety among patients, and ultimately contributing to improved health care outcomes for all.

## Conclusions

There is no doubt that research and academic institutions work with the intended purpose of seeing the eventual elimination of cardiovascular disparities altogether. However, intention and careful description of the disparities alone are *necessary but insufficient* to achieving our goal. We must remain accountable to the evidence and use what we know of disparities to change how we research, what we research, and what we do after research. As we invite community to improve health behaviors, we must also challenge ourselves to step away from the observation window and into the mirror to identify ways in which researchers might move beyond admiring the data and translate inequitable findings into sustainable community action.

## Funding support and author disclosures

The authors have reported that they have no relationships relevant to the contents of this paper to disclose.
